# Age-Related Changes in the Primary Motor Cortex of Newborn to Adult Domestic Pig *Sus scrofa domesticus*

**DOI:** 10.3390/ani11072019

**Published:** 2021-07-06

**Authors:** Salvatore Desantis, Serena Minervini, Lorenzo Zallocco, Bruno Cozzi, Andrea Pirone

**Affiliations:** 1 Department of Emergency and Organ Transplantation (DETO), University of Bari Aldo Moro, 70010 Valenzano, Italy; salvatore.desantis@uniba.it (S.D.); serenaminervini@gmail.com (S.M.); 2 Department of Pharmacy, University of Pisa, 56126 Pisa, Italy; l.zallocco@gmail.com; 3 Department of Comparative Biomedicine and Food Science, University of Padova, 35020 Legnaro, Italy; bruno.cozzi@unipd.it; 4 Department of Veterinary Sciences, University of Pisa, 56124 Pisa, Italy

**Keywords:** motor cortex, brain, swine, cytoarchitecture, calretinin, parvalbumin, growth

## Abstract

**Simple Summary:**

Over the past decades, the number of studies employing the pig brain as a model for neurochemical studies has dramatically increased. The key translational features of the pig brain are its size and similarities with the cortical and subcortical structures of other mammalian species; the brain seems to resemble a humans’ from an anatomical and histological point of view. Here we focus on the motor cortex during development, describing its cytoarchitecture and distribution of neural cells expressing two calcium-binding proteins: parvalbumin (PV) and calretinin (CR). PV and CR play an important role in the control of motor neuron output. The morphometric and immunohistochemical analysis of the present study revealed age-associated changes similar to those reported in the human motor cortex. These results confirm the pig brain is a valuable translational animal model during development.

**Abstract:**

The pig has been increasingly used as a suitable animal model in translational neuroscience. However, several features of the fast-growing, immediately motor-competent cerebral cortex of this species have been adequately described. This study analyzes the cytoarchitecture of the primary motor cortex (M1) of newborn, young and adult pigs (*Sus scrofa domesticus*). Moreover, we investigated the distribution of the neural cells expressing the calcium-binding proteins (CaBPs) (calretinin, CR; parvalbumin, PV) throughout M1. The primary motor cortex of newborn piglets was characterized by a dense neuronal arrangement that made the discrimination of the cell layers difficult, except for layer one. The absence of a clearly recognizable layer four, typical of the agranular cortex, was noted in young and adult pigs. The morphometric and immunohistochemical analyses revealed age-associated changes characterized by (1) thickness increase and neuronal density (number of cells/mm^2^ of M1) reduction during the first year of life; (2) morphological changes of CR-immunoreactive neurons in the first months of life; (3) higher density of CR- and PV-immunopositive neurons in newborns when compared to young and adult pigs. Since most of the present findings match with those of the human M1, this study strengthens the growing evidence that the brain of the pig can be used as a potentially valuable translational animal model during growth and development.

## 1. Introduction

The cerebral cortex mediates complex behaviors that are characteristic of mammals [1,2]. Voluntary movement through monosynaptic and polysynaptic control of skeletal muscles is mainly governed by the primary motor cortex (M1), otherwise known as Brodmann Area 4. 

Since the discovery of the motor cortex of the dog [3], the M1 was repeatedly mapped in a variety of animal species. Concerning the pig brain, the motor area has been localized between the cruciate sulcus and the coronal sulcus by means of histological, electrophysiological, and magnetic resonance imaging studies [4,5,6,7,8].

The cytoarchitecture of the M1 shows only five layers of the cerebral cortex, with the absence of layer four, and hence this motor region is often termed agranular. In addition, layer five is characterized by distinctive large pyramidal cells, which in primates are known as cells of Betz. In mammals such as sheep [9], cetaceans [10,11,12], and giraffes [13], these gigantopyramidal neurons do not appear to have the morphology typically seen for primate Betz cells in that they are much smaller. These large pyramidal cells are found in a peculiar, clustered arrangement in most mammals [4,9,10,11,12,13], except dogs [4]. The axons become the corticospinal or pyramidal tract and represent one of several descending influences on the motor neurons of the brain stem and spinal cord. The cytoarchitecture of the adult swine M1 is similar to other mammals. However, Campbell [4] reported “no real giant cells of Betz exist in the motor area of *Sus communis*”, and Bjarkam et al. [8] did not describe the presence of a clustered arrangement of layer five cells in the Göttingen minipig motor cortex. 

The neuronal population in the mammalian cerebral cortex mainly consists of two distinct groups: pyramidal neurons and interneurons. The former neurons are uniformly excitatory and use glutamate as a neurotransmitter. At the same time, the latter group is known as local circuit neurons, most of which are inhibitory, use γ-aminobutyric acid (GABA) as a principal neurotransmitter and play many important roles in cortical neuronal networks [14,15]. These inhibitory interneurons have been proposed to be important in the synchronization and rhythmic control of motor neuron output [16]. Based on the expression of three different calcium binding proteins (CaBPs), namely calbindin (CB), calretinin (CR), and parvalbumin (PV), it is possible to divide the cortical GABAergic interneurons into three largely non-overlapping populations [17]. These CaBPs are members of the EF-hand calcium-binding protein family [18]; they play a major role in buffering intracellular Ca^2+^, are involved in a variety of Ca^2+^-mediated signal transduction events [19,20,21,22], and are important for the precise timing and plasticity of synaptic events in neuronal networks [17 for reviews]. Moreover, it has been reported that CaBPs play a neuroprotective role in various pathological conditions by functioning as buffers for excess calcium [23].

The distribution of CaBPs has been described in many brain regions of several mammalian species [17,18,19,24,25,26,27,28,29,30,31,32,33,34,35,36,37,38,39]. Studies focused on the presence of CaBPs and positive neurons in the M1 cortex have been performed in rats [28], carnivores such as dogs and cats [29], horses [40], primates [2,40,41,42], including humans [23,30], but, to the best of our knowledge, not pigs. Little information is available on the early development of the pig brain, even at the cellular level [43]. In the present study, we compared the cytoarchitecture and distribution of CR- and PV-containing neurons in the M1 of newborn and adult pigs.

## 2. Materials and Methods

### 2.1. Tissue Samples

The brains were obtained from 27 female domestic pigs (*Sus scrofa domesticus*) used in the study of Minervini et al. [44]. The pigs were newborn (*n* = 4, weighting 1.18 ± 0.07 kg), young (*n* = 4, weighting 77.0 ± 3.0 kg, 5–6 months old), and adult (*n* = 19, weighting 158 ± 5.3 kg, greater than 10 months of age). Young and adult pigs were slaughtered at the slaughterhouse Maselli Industrie Srl (41°7′9.956″ N; 16° 28′29.975″ E), where animals were treated according to the European Community Council Regulation (CE1099/2009) concerning animal welfare during the commercial slaughtering process. Pigs were constantly monitored under mandatory official veterinary medical care. All the animals considered here were in good body condition and considered free of pathologies by the veterinary medical officer responsible for the health and hygiene of the slaughterhouse. Adult carcasses were put on the market. The brains of newborn piglets were sampled from animals found dead in the industrial farm and delivered to our facilities for rapid post-mortem diagnosis.

The three ages of the present experimental series represent newborn piglets, prepuberal individuals, and adult pigs. Age groups are based on the recognized stages of development and growth of the domestic pig according to the commercial process of pig farming and Reiland [45].

After removal from the skull, brains were fixed by immersion in 4% (*w*/*v*) phosphate-buffered (PBS) paraformaldehyde at 4 °C.

### 2.2. Localization of Motor Cortex and Tissue Preparation

**Figure 1:**
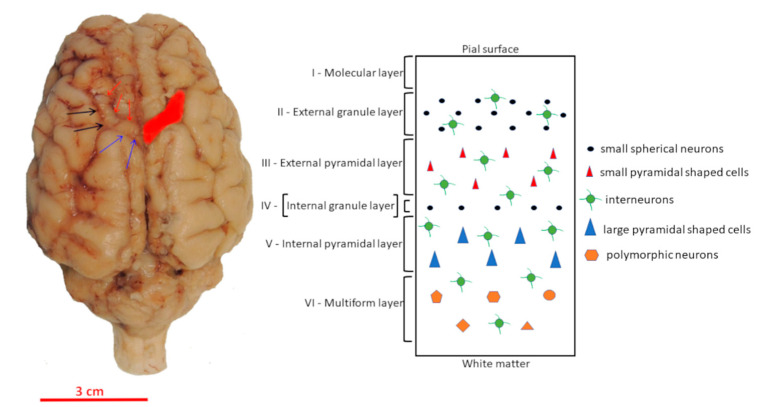
Dorsal view of the pig brain showing the localization of the primary motor cortex (red zone). Red arrows, sulcus cruciatus; blue arrows, sulcus ansatus; black arrows, sulcus coronalis. Scale bar: 3 cm. The drawing on the right shows the cytoarchitecture of the motor cortex, which has essentially no layer four, called the agranular cortex. Interneurons expressing CaBPs are scattered throughout the different layers.

The motor cortex was identified based on the position of the sulcus cruciatus and the sulcus coronalis [5,6,7] (Figure 1) and then removed.

The samples were extensively washed in PBS and embedded in paraffin wax. Coronal sections (5 μm thick) were serially cut and mounted on poly-l-lysine-coated glass slides. After de-waxing with xylene and hydrating in an ethanol series of descending concentrations, sections from all samples were stained with the Toluidine blue Nissl method for morphometric analysis. In contrast, sections from four animals for each age group were used for the immunohistochemical detection of CR and PV cells. 

### 2.3. Western Blot

After brain extraction, the motor cortex of adult pigs were sampled and immediately stored at −85 °C. Approximately 0.2 g of cerebral cortex tissue was suspended in 1.5 mL (7.5 *w*/*v*) of RIPA buffer (150 mM NaCl, 50 mM Tris-HCl pH 8, 1% IGEPAL CA-630, 0.1% sodium dodecyl sulfate) and placed into a pre-cooled potter and homogenized. After stirring and sonication (1 min, 3 times), samples were allowed to solubilize for 1 h at room temperature (RT) with occasional stirring. Thereafter, samples were centrifuged at 14,000× *g* for 20 min at 4 °C, and the resulting supernatants were transferred to new Eppendorf tubes. The protein concentration was determined using the Bio-Rad RC/DC-protein assay (Bio-Rad, Hercules, CA, USA), with bovine serum albumin (BSA) as a standard. Aliquots of cortex samples corresponding to 20 µg (for detection of CR) and 80 μg (for detection of PV) of proteins were mixed with Laemmli solution, loaded onto 8–16% polyacrylamide gel (TGX, Bio-Rad), separated, and transferred to polyvinylidene difluoride (PVDF) membranes (0.2µm) using a Trans blot turbo apparatus (Bio-Rad). After blotting, PVDF membranes were incubated at 50 °C for 30 min and then blocked with 3% low-fat dried milk and 0.2% (*v*/*v*) Tween 20 in Tris-Buffered Saline for 1 h at RT. Subsequently, membranes were incubated with a primary antibody (CR, 1:500, Abcam, Cambridge, UK, ab702; PV, 1:1000, Sigma-Aldrich, Saint Louis, MO, USA, details reported in Table 1) for 2 h at RT. After washing, membranes were incubated with a secondary antibody for 1 h at RT. HRP-conjugated goat anti-mouse (1:10,000 dilution; PerkinElmer, Waltham, MA, USA) and anti-rabbit (dilution 1:10,000, Enzo Lifesciences, New York, NY, USA) (details reported in Table 2) were used as secondary antibodies. The detection of the immunoreactive band was performed using ECL-Pro (Perkin Elmer, Waltham, MA, USA) and Image Quant LAS4010 (GE HealthCare, Chicago, IL, USA).

### 2.4. Immunohistochemistry

Immunoperoxidase reaction was performed on serial paraffin sections (5 μm) using a rabbit polyclonal anti-CR (1:100, Abcam, ab702) and a mouse monoclonal anti-PV (1:2000, Sigma-Aldrich, P3088, Clone PARV-19) (details reported in Table 1). Epitope retrieval was carried out at 120 °C in a pressure cooker for 5 min with a Tris/EDTA buffer, pH 9.0. Sections were pretreated with 1% H_2_O_2_ in PBS for 10 min to quench endogenous peroxidase activity, then rinsed with 0.05% Triton-X (TX)-100 in PBS (3 × 10 min) and blocked for 1 h with 5% normal horse serum (NHS) (PK-7200, Vector Labs, Burlingame, CA, USA) in PBS. Serial sections were incubated overnight at 4 °C in a solution containing the anti-CR or anti-PV with 2% normal horse serum 0.05% TX-100 in PBS. Sections were then rinsed in PBS (3 × 10 min), followed by incubation with biotinylated anti-mouse (BA-2001, Vector Labs) or anti-rabbit IgG (BA-1100, Vector Labs) (details reported in Table 2), and then with ABC reagent (Vectastain Kit, PK-7200, Vector Labs). Sections were again rinsed in PBS for 3 × 10 min. Staining was visualized by incubating the sections in diaminobenzidine (sk-4105, Vector Labs) solution. The specificity of immunohistochemical staining was tested by replacing either the primary antibodies, anti-mouse IgG, or the ABC complex with PBS or non-immune serum. Under these conditions, staining was abolished. Furthermore, according to the manufacturing details, monoclonal anti-PV reacts with parvalbumin originating from a pig. In contrast, pig calretinin (https://www.ncbi.nlm.nih.gov/protein/NP_001181909.2 (accessed on 3 May 2021) has a very high (97%) percent identity with the calretinin of the species that the polyclonal anti-CR reacts with (see manufacturing details). In addition, the specificity of the antibodies had already been tested in previous studies (RRID code Table 1). Furthermore, positive controls were performed on mouse cerebellum (Supplementary File S1) and pig cerebellum (Supplementary File S2).

### 2.5. Double Immunofluorescence

Calretinin/parvalbumin co-localization was carried out on two coronal sections (50 μm spaced) of M1 for each brain. In each section, five micrograph fields from the whole region of M1 were randomly detected using a 20× magnification and analyzed for possible CR/PV co-expression. 

Immunofluorescent reactions were performed using a mouse monoclonal anti-Parvalbumin (PV) and a rabbit polyclonal anti-calretinin (CR) (details reported in Table 1). Epitope retrieval was carried out at 120 °C in a pressure cooker for 5 min with a Tris/EDTA buffer, pH 9.0. Sections were then blocked for 1 h with 5% NHS (PK-7200, Vector Labs), 0.05% TX-100 in PBS, and incubated overnight at 4 °C in a solution containing the anti-PV and the anti-CR diluted in 1% NHS in PBS. Sections were then rinsed in 0.1 M PBS (3 × 10 min) and incubated with a DyLight 488 anti-rabbit IgG and DyLight 549 anti-mouse IgG for 1 h at room temperature (details reported in Table 2). Finally, sections were washed with PBS and cover-slipped with Vectashield with DAPI (H-1500, Vector Labs). The negative control was performed by replacing the primary antibodies with 1% NHS in PBS. Under this condition, staining was abolished. 

Microphotographs were collected under a Nikon Ni-e light microscope (Nikon Instruments Spa, Calenzano, Italy) with Nikon Plan lens, fully equipped for fluorescence acquisition connected to a personal computer via a Nikon digital image processing software (Digital Sight DS-U1, NIS-Elements BR-4.13.00 software).

### 2.6. Morphometry and Statistical Analysis

Cytoarchitecture and layering were assessed in six representative Nissl-stained coronal sections (one 5 μm serial section every 10th section) of each brain. The M1 thickness was measured on 50 micrograph fields randomly detected using a 4× magnification, whereas the cell density was estimated in the same fields using photos taken with a 10× objective lens. The cell sizes of large pyramidal neurons in layer five were measured on twenty cells using microphotograph fields randomly detected and taken at 100× magnification.

Calretinin- and parvalbumin-immunoreactive (ir) neuron count was carried out on three coronal sections of M1 of each brain. In each section, the whole area of the M1 cortex was analyzed at 10×, and the number of positive cells was counted with a 20× objective to better detect positive neurons. To compare immunoreactive neuronal subpopulations containing CR and PV, the neuronal density was estimated as the number of cells per mm^2^. All photos were taken with a light photomicroscope Eclipse Ni-U (Nikon, Tokyo, Japan) equipped with a digital camera (DS-U3, Nikon). The images were analyzed by the image-analyzing program NIS Elements BR (Vers. 4.20) (Nikon). 

Values were expressed as means ± standard deviation (S.D.). Data were statistically analyzed by ANOVA using the Statistical Package for Social Science (SPSS, version 19) software. The differences of the means were defined using the Bonferroni test and considered significant when *p* < 0.05.

## 3. Results

### 3.1. Western Blot

**Figure 2:**
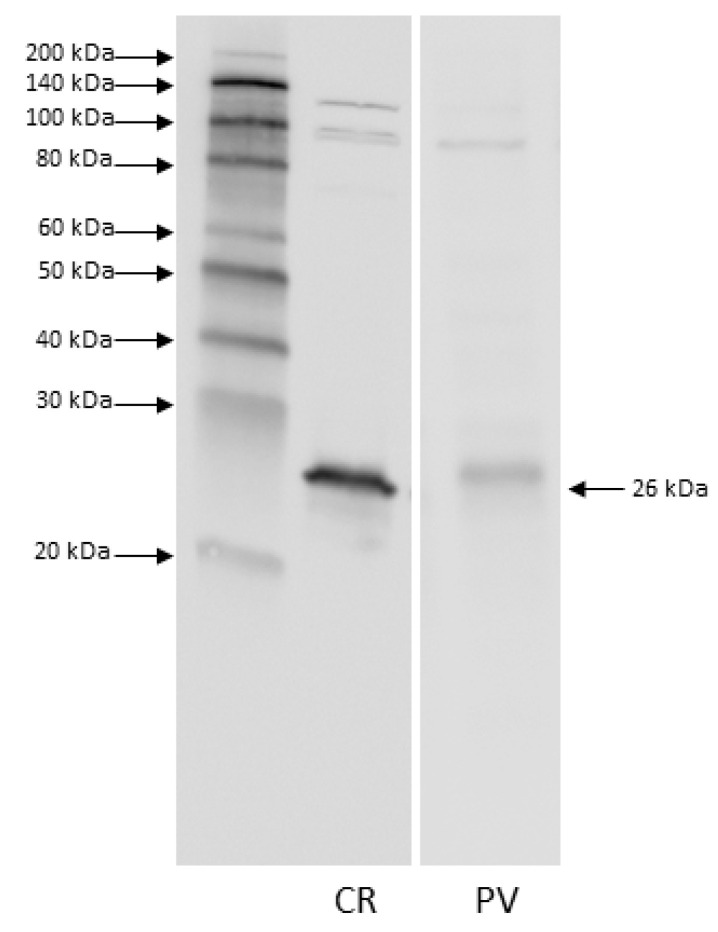
Immunoblot analysis revealed the presence of both parvalbumin (PV) and calretinin (CR) in the motor cortex. A single immunoreactive band at about 26 KDa was detected for anti-CR and for anti-PV, the latter probably representing a dimeric form of PV.

Immunoblot analysis was performed to evaluate the presence of PV and CR in the pig motor cortex and to test the specificity of the commercial antibodies used. A single immunoreactive band was found to be approximately 26 kDa for both anti-CR and anti-PV (Figure 2). The anti-PV band probably represents a dimeric form of the protein.

### 3.2. Morphology

**Figure 3:**
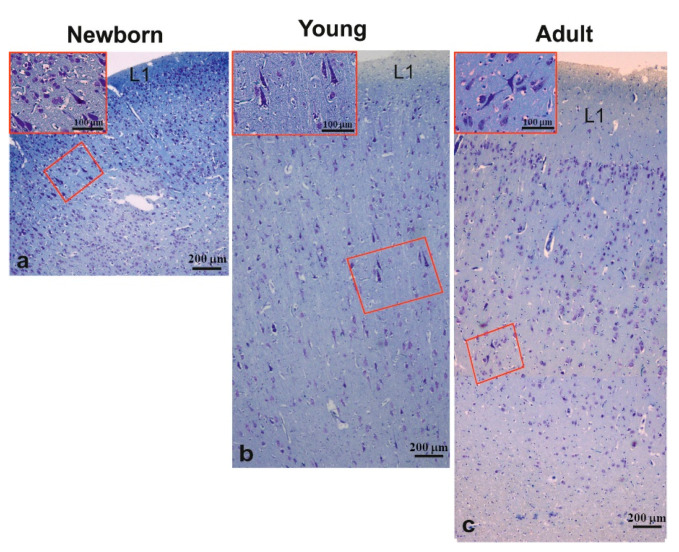
Nissl staining of the motor cortex of newborn (**a**), young (**b**), and adult (**c**) pigs. Insets of (**a**–**c**) show a high magnification of large pyramidal neurons in squared area of (**a**–**c**) pictures. L1, layer one.

Sections stained with Nissl staining displayed a different organization of the M1 from neonates compared with young and adult M1s. Neonate M1s showed a dense neuronal arrangement which made the discrimination of the typical six cell layers difficult, except for layer one (Figure 3). As for M1 of young and adult pigs, it did not exhibit a well distinguishable layer four (Figure 3b,c).

The morphometric data of M1 are summarized in Table 3. The morphometric analysis revealed that M1 cell density decreased with age, being significantly higher (*p* < 0.001) in newborns (425.71 ± 68.40/mm^2^) than young (270.19 ± 33.51/mm^2^) or adults (251.33 ± 28.01/mm^2^); although, no statistical difference was observed between the two latter samples. Moreover, M1 thickness showed an age-related increase being significantly (*p* < 0.001) thinner in newborns (1250.39 ± 41.04 µm) than in young (1962.3 ± 48.12 µm) or adult (2058.12 ± 37.27 µm) pigs, which did not display a statistical difference. Concerning the thickness of layer one, there was a significant (*p* < 0.001) progressive age-related increase measuring 135.14 ± 21.9 µm in neonates, 264.73 ± 19.76 µm in young, and 438.33 ± 50.12 µm in adult animals.

The largest pyramidal neurons were detected in the middle layer of the M1 in neonates (Figure 3a) and in layer five of young and adult M1s (Figure 3b,c). These neurons, which were arranged in clusters of two to three cells, did not show an age-related size change, which ranged from 33 to 34 µm (Table 3). 

### 3.3. Immunohistochemistry

#### 3.3.1. Calretinin

**Figure 4:**
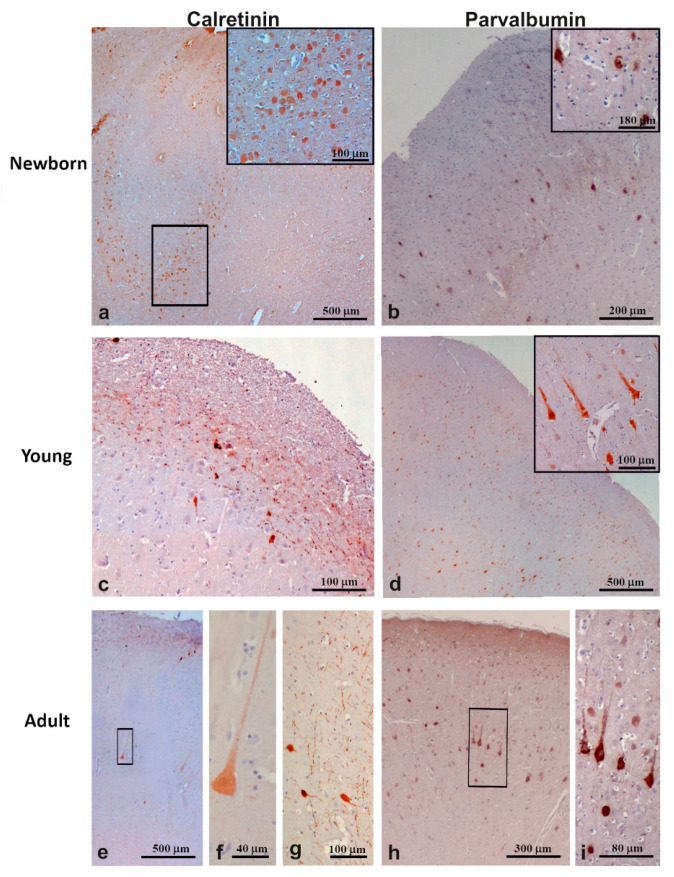
Immunocytochemistry of calcium-binding proteins in the motor cortex (M1) of newborn (**a**,**b**), young (**c**,**d**), and adult (**e**–**i**) pigs. Note the network of CR-ir cells in the outer layer of M1 from young and adult pigs (**c**,**g**)**.** PV-ir cells of M1 from newborns (inset of **b**) display a different shape compared to one from young (inset of **d**) or adult (**i**) pigs. Inset of (**a**) is a high magnification of the squared zone. (**f**,**i**) display high magnifications of squared zones in (**e**,**h**), respectively.

Calretinin-ir neurons were found in the outer and inner layers of M1 from newborn pigs (Figure 4a) and in the outer layers of M1 of young and adult pigs (Figure 4c,e). In newborns, CR-ir neurons showed round or fusiform somata with one, or occasionally two, processes (inset of Figure 4a). The M1 of young and adult pigs displayed an extensive network of CR-ir axons from bipolar cells (Figure 4c,g), although rare CR-positive neurons with pyramidal-like somata were also found in the inner layer (Figure 4e,f). The density of CR-ir neurons (Table 3) was higher in newborns than in the young or adults (25 cells/mm^2^ vs. 16 cells/mm^2^), whereas the cell size did not show statistical difference being 31.18 ± 7.47 µm in newborns, 30.65 ± 4.39 µm in young, and 37.01 ± 8.55 µm in adults.

#### 3.3.2. Parvalbumin

Parvalbumin-ir neurons were scattered in all layers of the M1 except layer one of the investigated pigs (Figure 4b,d,h). PV-ir cells were more numerous in newborns (27.44 ± 2.98 cells/mm^2^) than in the young or adults (17.86 ± 4.22 cells/mm^2^ and 19.55 ± 4.67 cells/mm^2^, respectively) (Table 3). Morphometric analysis revealed that the diameter of PV-ir cells was similar in the M1 of all samples, although a non-significant age-related increasing trend was observed: 34.93 ± 7.96 µm in newborns, 35.11 ± 19.73 µm in young, and 36.40 ± 8.03 µm in adults. PV-containing neurons were multipolar in shape, although pyramidal PV-ir neurons were also observed in deep layers of M1 from young and adult pigs (Figure 4 inset of d,i).

#### 3.3.3. Double Immunofluorescence

**Figure 5:**
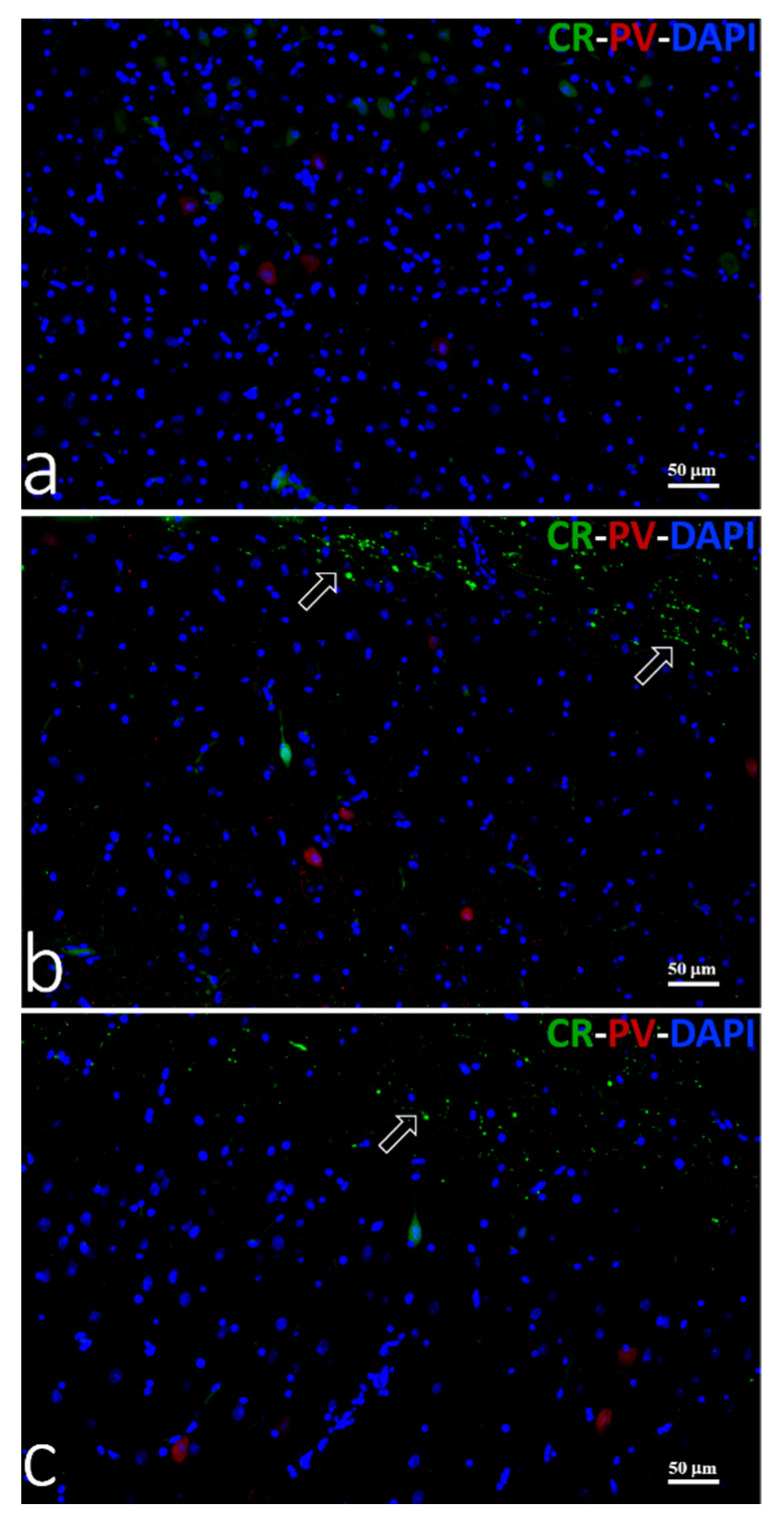
Immunofluorescent staining to PV (red) and CR (green) in M1. Neurons co-expressing PV and CR were not found in the motor cortex of newborn (**a**), young (**b**), or adult (**c**) domestic pig. Arrows (**b**,**c**) indicate CR positive varicose fibers in layers I/II of M1. Cell nuclei were counterstained with DAPI.

In all analyzed fields of M1, PV and CR co-expression were not seen in newborn, young, or adult pigs (Figure 5a–c). 

## 4. Discussion

This study compares the cytoarchitecture and distribution of CR- and PV-containing neurons in the M1 of newborn, prepuberal, and adult pigs.

Moreover, we investigated the possibility of a co-localization between PV and CR. To this end, we conducted a double immunofluorescence study which showed that the two proteins were not co-expressed in M1 during brain development. This was in agreement with that reported by Tremblay et al. [46].

The specificity of the primary antibodies employed was tested by performing Western blot analysis, which revealed the presence of both CR and PV in the pig motor cortex. 

The molecular masses of CR and PV were detected at about 29 KDa and 12 KDa, respectively [47]. The PV band at 26 KDa we observed probably represented a dimeric form of this protein that had already been reported in fish [48,49]. However, to further test the anti-PV specificity, we immunostained the pig cerebellar cortex as a well-established positive control brain structure. The immunolocalization patterns we found for PV (S2) were consistent with what was formerly reported by Bastianelli [50] and Schmidt et al. [51].

The M1 of the domestic swine was localized between cruciate and coronal sulci [5,6,7] and characterized by the lack of an evident layer four. This specific cytoarchitectonic feature is shared by Göttingen minipigs [8], other ungulates [9,13,40,52], and cetaceans [12,53]. The morphometric analysis revealed significant aging-dependent changes in thickness and neuronal cell density. Specifically, the M1 thickness increased with age: 1250.39 ± 41.04 µm in newborns, 1962.23 ± 48.12 µm in young, and 2058.12 ± 37.27 µm in adults. This result agrees with the findings in human M1 by Amunts et al. [54], which detected an increase in the width of M1 during early postnatal ontogeny. The thickness of the M1 of adult domestic pigs is comparable to that of most terrestrial mammals (for comparison, see [52]), but noticeably higher than that of Göttingen minipigs, which measure about 1600 µm in one-year-old animals [8]. In the current study, we found that, unlike the thickness, the cell density (the number of neurons/mm^2^ of M1) decreased from birth to one year of age. The reduction was high (36%) between newborn and young pigs and low (7%) but not statistically significant between young and adult pigs. Since massive postnatal neurogenesis does not occur in the neocortex of the domestic pig [55], the results we found could depend on the glial cell proliferation, dendritic and axonal arborization, as well as the increased myelination. It has been observed that the pig brain, like that of humans, develops perinatally, with a brain growth spurt extending from mid-gestation to early postnatal life [56,57]. Particularly, a large increase in postnatal brain growth of domestic pigs from 2 to 12 weeks of age occurs when the growth rate began to slow down [58,59]. Therefore, similar to humans, most neurogenesis and migration occur early in the prenatal period, with astrocyte and oligodendrocyte proliferation extending from the late prenatal to the postnatal period [60]. 

In the present study, we detected a significant age-related increase in the thickness of layer one, which is heavily innervated by fibers from lower layers and axon collaterals from other neocortical areas and the thalamus [61]. In human M1, layer one is the first level of the cortex to develop and to achieve maturity; its development and maturation are related to and seem to follow the early arrival of afferent fibers to this level of the cortex [62]. Since layer one activity is vital for learning and processing information in neocortical circuitry [63], the observed increase in layer one thickness could be related to the age-related experience. Very recently, it has been demonstrated that learning-related plasticity in the human motor cortex is associated with learning-related changes in the somatosensory cortex [64], which has repercussions on subsequent movements as a result of experience-dependent cognition.

The immunohistochemistry revealed that the morphology of CR-ir neurons in pig M1 changes with age. CR-ir neurons were round or fusiform in M1 of newborns and bipolar in young and adult pigs. Moreover, M1 of the latter two samples also contained rare CR-positive pyramidal somata in the deep layers. Bipolar or fusiform calretinin-positive cells, resembling the Cajal-Retzius cells, have been detected in layer one of the prefrontal, temporal, parietal, and occipital neocortical areas of newborn, young, and adult domestic pigs [32]. Unfortunately, no data concerning CR-ir neurons in M1 are present in the cited study. However, interspecific morphological differences in CR-containing neurons have been reported in the literature. CR-ir neurons displayed round or fusiform and bipolar shapes in owl monkey M1 [42], and double bouquet type, or bipolar morphologies in human M1 [23]. Moreover, typical CR-ir pyramidal neurons were observed in dogs and, less abundantly, in cat M1 [29]. The evaluation of the CR-ir neuron density revealed an age-dependent reduction of these cells in pig M1. Specifically, a large decrease (approximately 37%) was detected between the one-day-old and six-month-old animals. After this age, no significant changes were observed. A similar trend has been observed in the number of calretinin-positive neurons of the pig prefrontal cortex, which decreases 25 % in the first quarter of life; whereas, they did not undergo any significant changes between three-month-old and one-year-old animals [32]. An age-related decrease of CR-ir neurons has also been detected in the motor cortex of rats [65], whereas no significant changes in CR-ir neuron numbers have been observed in human M1 when young and old brains were compared [30]. 

PV-ir neurons were observed in layers II, III, V, and VI of the pig M1. A similar pattern has been observed in the M1 of non-human primates such as chimpanzees and macaque [40] and in the somatosensory cortex of rodents (mice, rats, and gerbils) [37]. In human M1, PV-containing cells have been mainly detected in layers two to four [23]. A large presence of multipolar PV-containing neurons was observed in all of our investigated specimens. This type of PV-positive cell has also been detected in cetaceans and Cetartiodactyls [29]. In the current study, we also found pyramidal PV-ir neurons in the deep layers of M1 of young and adult pigs. The presence of PV-containing pyramidal neurons has been observed in layer five of the primary motor cortex of adult anthropoid primates [41]. Morphological variation within PV-containing neurons among species has been reported. For example, chandelier cells containing PV are present in primate M1 [66] but not in canids [67]. In addition, van Brederode et al. [28] found that PV-ir cells are represented by several classes of non-pyramidal cells in the motor area of the rat. It has been reported that the various morphological classes of PV-ir cells could regulate the rhythmic oscillations of pyramidal cell populations and have been identified as fast-spiking based on their brief action potentials and the absence of spike adaptation (reviewed by [2]). In the current study, the number of PV-ir neurons showed the same trend observed for CR-ir neurons. PV-containing neurons decrease (approximately 35%) between the newborn and six-month-old animals, and they remained unchanged between young and adult pigs. To the best of our knowledge, data concerning the density of PV-containing neurons in the M1 of mammalian newborns are lacking. A previous study has demonstrated that the density of PV-ir neurons did not show an age-related change in M1 of humans between 26 and 93 years old [30]. Compared to data shown in Figure 1 by Bu et al. [30], the density of PV positive neurons in M1 from young and adult pigs is approximately 30% lower than young and adult human M1. This may reflect a lower regional specialization in interneuron circuitry of pigs compared to humans. However, it has been reported that the number of PV-ir neurons in the prefrontal cortex was not significantly different between young and aged canines [68], whereas it tends to increase with age in the somatosensory cortex of rodents [37].

## 5. Conclusions

In conclusion, this study demonstrates that the pig M1 presents age-associated changes characterized by (1) thickness increase and neuronal density reduction during the first year of life; (2) morphological changes of CR-ir neurons in the first months of life; (3) higher density of CR-ir and PV-ir neurons in newborns when compared to young and adult pigs. Our data confirm that the motor cortex of the domestic pig can be considered a translational model to simulate the condition of the human adolescent brain [69].

## Figures and Tables

**Table 1:** Primary Antibodies.

Antibody	Immunogen	Manufacturing Details	Dilution
Anti-PV	PARV-19 hybridoma produced by the fusion of mouse myeloma cells and splenocytes from an immunized mouse. Purified frog muscle parvalbumin was used as the immunogen	Sigma-Aldrich, mouse monoclonal, Clone PARV-19, Product No. P 3088 RRID: AB477329	1:2000
Anti-CR	Full length protein	abcam, rabbit polyclonal, ab702 RRID: AB305702	1:100

**Table 2:** Secondary Antibodies.

Antibody	Type	Manufacturing Details	Dilution
Biotinylated	Anti-mouse IgG (H + L)	Vector Labs, Burlingame, horse, Cat.n. BA-2001, Lot.n. ZC1230 RRID: AB2336180	5 µg/mL
Biotinylated	Anti-rabbit IgG (H + L)	Vector Labs, Burlingame, horse, Cat.n. BA-1100, Lot.n. ZA0319 RRID: AB2336201	5 µg/mL
DyLight 549	Anti-mouse IgG (H + L)	Vector Labs, Burlingame, horse, Cat.n. DI-22549, Lot.n. Z0416	10 µg/mL
DyLight 488	Anti-rabbit IgG (H + L)	Vector Labs, Burlingame, horse, Cat.n. DI-1088, Lot.n. Z1005	10 µg/mL
HRP conjugate	Anti-rabbit IgG	Enzo life science, goat, Cat.n. ADI-SAB-300J	1:10,000
HRP conjugate	Anti-mouse IgG	Perkin Elmer, goat, Cat.n. NEF822	1:10,000

**Table 3:** Data concerning the primary motor cortex of the female domestic pig *Sus scrofa domesticus*.

Parameters	Age (Days)			
	1 (*n* = 4)	150–180 (*n* = 4)	>300 (*n* = 19)	*p*-Value
Brain weight (g)	33.97 ± 1.99 ^a^	126.21 ± 7.5 ^b^	142.30 ± 6.22 ^c^	<0.001
Cell density (n/mm^2^)	425.71 ± 68.40 ^a^	270.19 ± 33.51 ^b^	251.33 ± 28.01 ^b^	<0.001
M1 thickness (µm)	1250.39 ± 41.04 ^a^	1962.23 ± 48.12 ^b^	2058.12 ± 37.27 ^b^	<0.001
Layer 1 thickness (µm)	135.14 ± 21.9 ^a^	264.73 ± 19.76 ^b^	438.33 ± 50.12 ^c^	<0.001
Large pyramidal neurons size in layer 5 (µm)	33.21 ± 4.21	34.82 ± 3.04	34.68 ± 3.13	0.359
CR-ir density (n/mm^2^) *	25.04 ± 3.16 ^a^	15.78 ± 2.90 ^b^	16.51 ± 2.02 ^b^	<0.001
PV-ir density (n/mm^2^) *	27.44 ± 2.98 ^a^	17.86 ± 4.22 ^b^	19.55 ± 4.67 ^b^	<0.001

* Four animals for each age group were used for the detection of calretinin and parvalbumin cells. Different letters in the same row denote significant differences.

## Data Availability

The data presented in this study are available on request from the corresponding author.

## Supplementary Materials

The following are available online at https://www.mdpi.com/article/10.3390/ani11072019/s1, File S1: Positive controls showing immunoperoxidase staining of Parvalbumin and Calretinin on mouse brain sections. File S2: Positive control showing immunoperoxidase staining of Parvalbumin on pig cerebellum sections.
