# Supplementary material for: Age-Related Changes in the Primary Motor Cortex of Newborn to Adult Domestic Pig Sus scrofa domesticus
Source: Animals (Basel). 2021 Jul 6;11(7):2019. doi: 10.3390/ani11072019 (PMC8300406; doi:10.3390/ani11072019)
Supplement: Supplementary file 1 [file animals-11-02019-s001.zip › File S1.pdf]

Parvalbumin

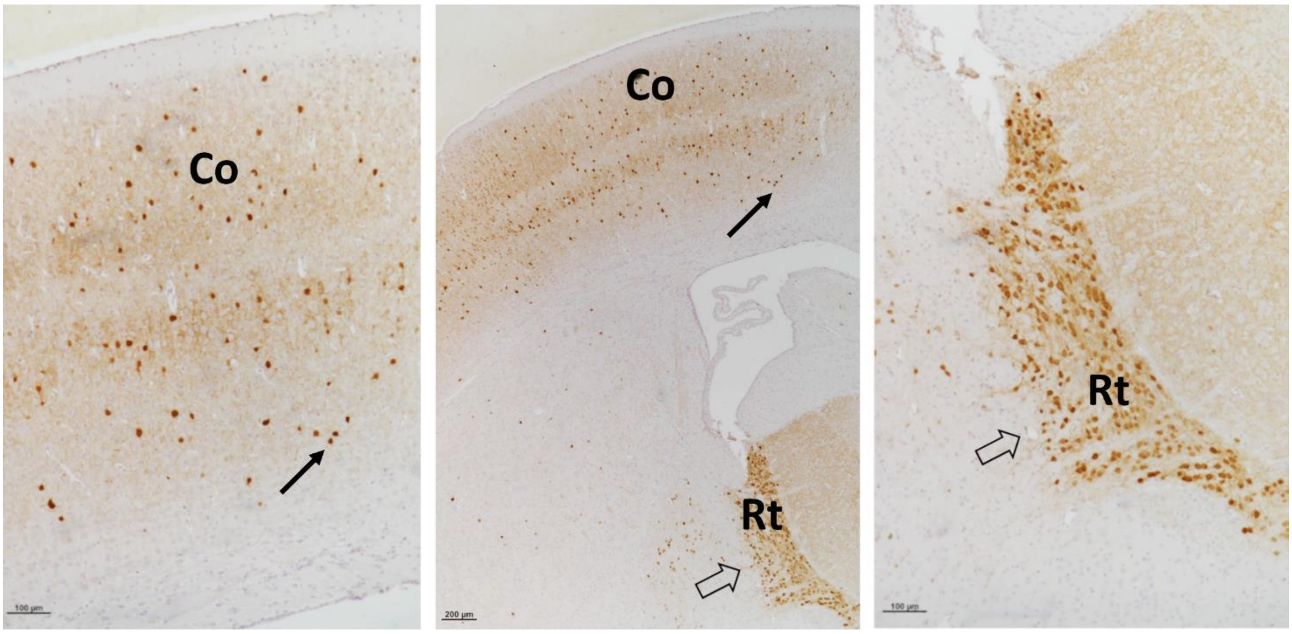

Immunoperoxidase staining of mouse brain sections. PV immunolabeled neuronal somata were observed in the cortex (Co) and in the reticular thalamic nucleus (Rt).

Calretinin

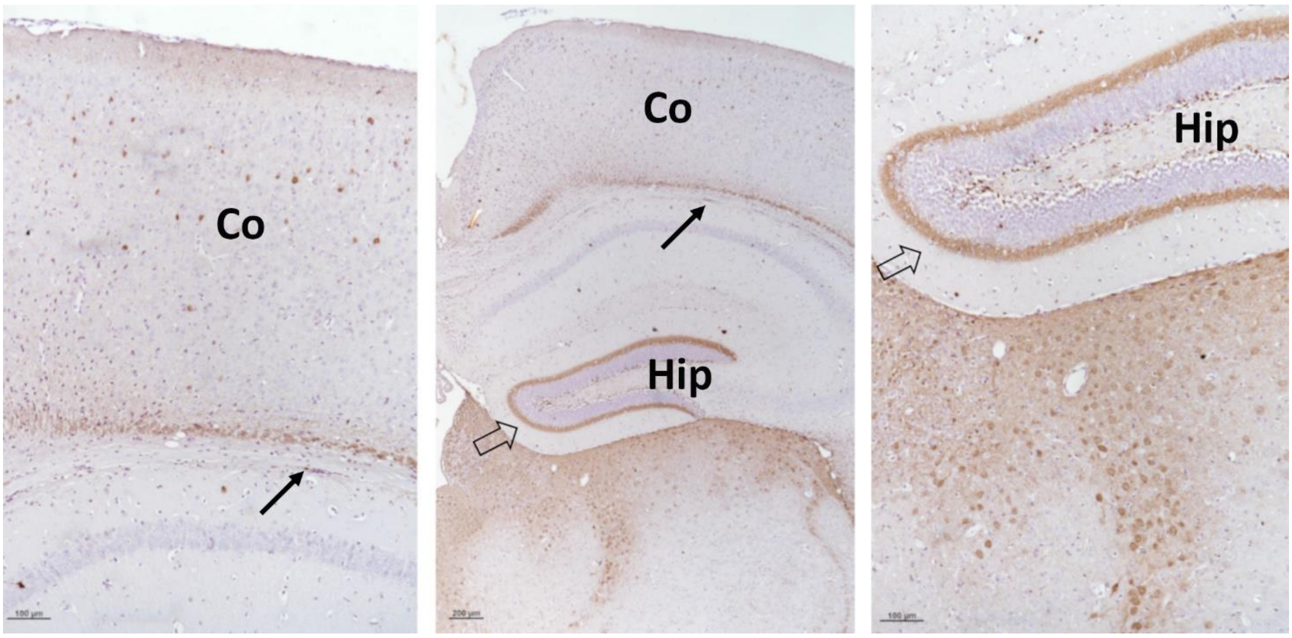

Immunoperoxidase staining of mouse brain sections. CR immunolabeling was found in neuronal somata of the cortex (Co) and in the dentate gyrus of the hippocampus (Hip).
