# Supplementary material for: Age-Related Changes in the Primary Motor Cortex of Newborn to Adult Domestic Pig Sus scrofa domesticus
Source: Animals (Basel). 2021 Jul 6;11(7):2019. doi: 10.3390/ani11072019 (PMC8300406; doi:10.3390/ani11072019)
Supplement: Supplementary file 1 [file animals-11-02019-s001.zip › File S2.pdf]

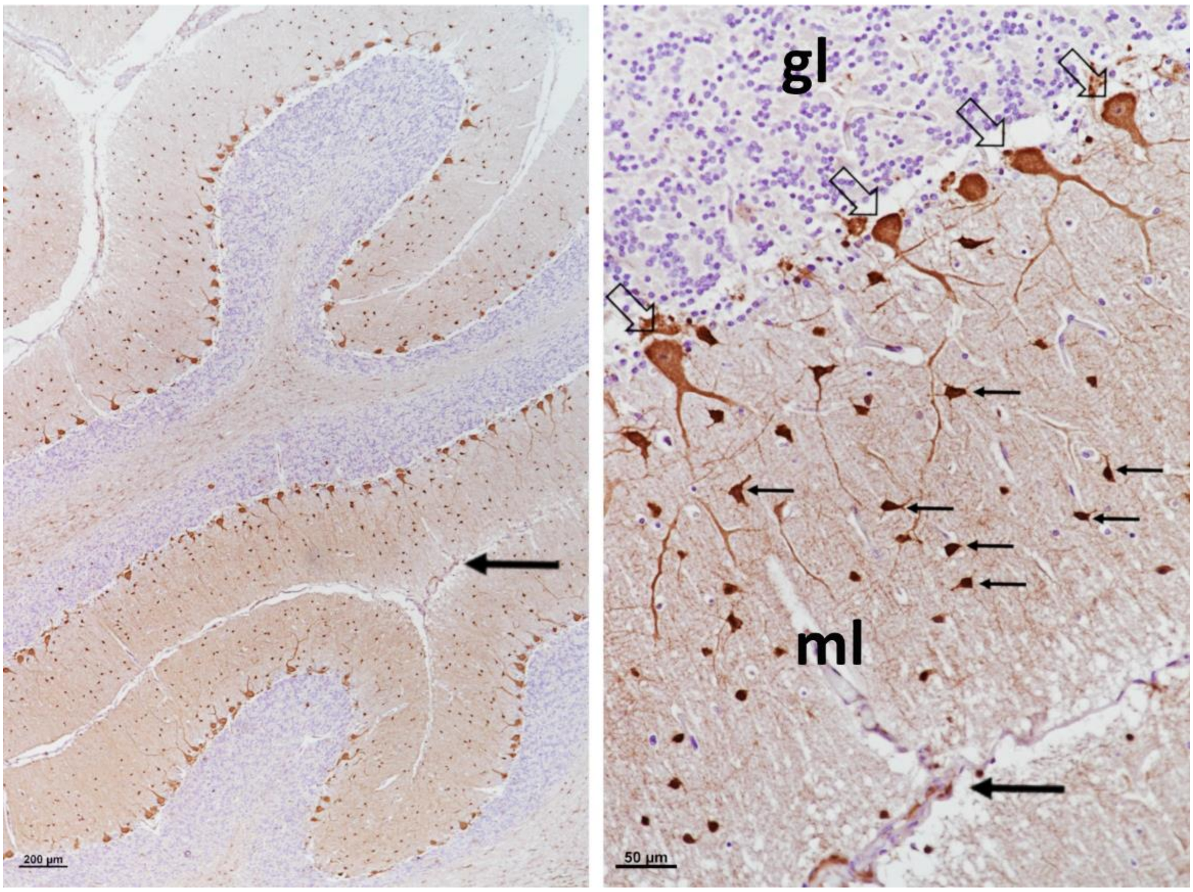

Immunoperoxidase staining of pig cerebellum sections. PV immunolabeling characterized the soma and dendrites of Purkinje cells (empty arrows); immunoreactivity was also seen in the stellate cells (small arrows) within the molecular layer (ml). gl, granular layer.
